# Increased Risk of Tooth Loss in Postmenopausal Women With Prevalent Vertebral Fractures: An Observational Study

**DOI:** 10.1002/jbm4.10822

**Published:** 2023-09-28

**Authors:** Akira Taguchi, Tomohiko Urano, Yukio Nakamura, Masataka Shiraki

**Affiliations:** ^1^ Department of Oral and Maxillofacial Radiology Matsumoto Dental University Shiojiri Japan; ^2^ Department of Geriatric Medicine International University of Health and Welfare School of Medicine Narita Japan; ^3^ Department of Orthopaedic Surgery Shinshu University School of Medicine Matsumoto Japan; ^4^ Department of Internal Medicine Research Institute and Practice for Involutional Diseases Azumino Japan

**Keywords:** DENTAL BIOLOGY, FRACTURE, MENOPAUSE, OSTEOPOROSIS, VERTEBRAE

## Abstract

The association between prevalent fractures and tooth loss in postmenopausal women remains unclear. Herein, we investigated the association between prevalent vertebral and nonvertebral fractures, the number of teeth present at baseline, and the number of teeth lost during follow‐up in postmenopausal Japanese women. This cross‐sectional study enrolled 843 participants (mean age 68.3 years). The number of teeth at follow‐up was evaluated in 655 women in this longitudinal study. The participants were divided into four groups according to their prevalent fracture status: no fractures, vertebral fractures alone, nonvertebral fractures alone, and both fracture types. After adjusting for covariates, Poisson regression analyses were performed to investigate differences in the number of teeth at baseline and that lost during the follow‐up period among the four groups. Participants with prevalent vertebral fractures alone had significantly fewer teeth at baseline than those in participants without fractures or nonvertebral fractures alone (*p* < 0.001 for both). Furthermore, they lost more teeth during the follow‐up period than did those with no fractures (*p* = 0.021) and tended to lose more teeth than did those with nonvertebral fractures alone or both prevalent fracture types. We observed no significant difference in the number of teeth lost between the participants with nonvertebral fractures alone and those with no fractures. Postmenopausal women with prevalent vertebral fractures may be at a higher risk of tooth loss. © 2023 The Authors. *JBMR Plus* published by Wiley Periodicals LLC on behalf of American Society for Bone and Mineral Research.

## Graphical abstract

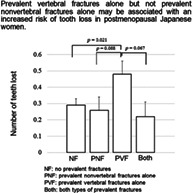

## Introduction

Loss of teeth may contribute to a higher risk of several systemic diseases such as cardiovascular diseases,^(^ ^1^ ^)^ lung cancer,^(^ ^2^ ^)^ and rheumatoid arthritis.^(^ ^3^ ^)^ Community‐dwelling older Japanese individuals with fewer teeth may be more likely to develop functional disabilities than those with 20 or more teeth.^(^ ^4^ ^)^ Fewer number of teeth may also be associated with a higher risk of mortality.^(^ ^5^ ^)^

In cross‐sectional and longitudinal studies, Krall and colleagues suggested an association between systemic bone and tooth loss in postmenopausal women.^(^ ^6^, ^7^ ^)^ Jang and colleagues reported that more remaining teeth were significantly associated with higher skeletal bone mineral density (BMD) in postmenopausal Korean women.^(^ ^8^ ^)^ Further, several studies have shown that osteoporosis‐preventing estrogen therapy has protective effects on tooth retention.^(^ ^9^, ^10^, ^11^ ^)^ Postmenopausal women with low BMD may be more susceptible to tooth loss than those with normal BMD. Several studies have evaluated the association between skeletal BMD and periodontal disease, which is a major cause of tooth loss, particularly in postmenopausal women. Hong and colleagues reported a significant association between periodontitis, osteoporosis, and fractures in women aged ≥53 years.^(^ ^12^ ^)^ Low skeletal BMD and osteoporosis in postmenopausal women may contribute to periodontal disease progression, resulting in an increased risk of tooth loss.

The potential association between tooth loss and low BMD indicates that a small number of teeth may be associated with an increased risk of fragility fractures. Several studies have evaluated the association between osteoporosis and tooth loss, particularly in postmenopausal women. However, in almost all studies, osteoporosis was determined using densitometry, but fractures—which represent an important outcome of osteoporosis—were not evaluated. Thus, limited data are available regarding the association between vertebral and nonvertebral fractures other than hip fractures and tooth loss in postmenopausal women.

Only three studies have evaluated the association between osteoporotic fractures and tooth loss.^(^ ^13^, ^14^, ^15^ ^)^ In a prospective study of Japanese male dentists, Wakai and colleagues reported a weak correlation between tooth loss and a higher risk of subsequent hip fractures.^(^ ^13^ ^)^ Similarly, Yu and colleagues reported that the presence of an additional tooth was associated with a slightly lower risk of self‐reported hip fractures.^(^ ^14^ ^)^ In their study, approximately half of the participants were men aged ≥20 years. Martínez‐Maestre and colleagues reported that postmenopausal women with prevalent spine fractures determined using radiography had a significantly lower number of teeth than those of individuals without spine fractures;^(^ ^15^ ^)^ the median numbers of teeth were 19 and 23 in the fracture and nonfracture groups, respectively (*p* = 0.007). However, their sample size was too small and the participants were limited to postmenopausal women with at least 10 teeth.

This study aimed to investigate the association between prevalent vertebral and nonvertebral fractures and the number of teeth at baseline and that lost during the follow‐up period.

## Materials and Methods

### Ethical considerations

The Ethics Committee of the Research Institute and Practice for Involutional Diseases, Japan, reviewed the study protocol in 1993 for the first version and in 2000 for the present version (approval date April 30, 2000). The study adhered to the principles of the Declaration of Helsinki. All participants gave written informed consent.

### Study participants

The Nagano Cohort Study was initiated in 1993 at a primary care institution in Nagano Prefecture, Japan. Postmenopausal and perimenopausal women were included in this study. The participant registration is ongoing, and almost 4000 women have been registered to date. The present study was conducted on a subset of participants registered in the Nagano Cohort Study. Specifically, the dental project registered consecutive patients who visited a primary care institution between January 5, 2010, and December 25, 2017. The participants were followed up annually and the teeth were counted. The observation period for the dental project ended on December 25, 2018.

The participant inclusion and exclusion criteria are described in our recent study.^(^ ^16^ ^)^ For the cross‐sectional study using baseline data, a trained physician counted the number of teeth present and collected information on periodontal disease and the daily number of toothbrushing sessions for 843 participants. The data evaluated in this study were obtained from patient characteristics at registration. Of 843 participants, 104 edentulous women were excluded from the longitudinal study. Eighty‐four women did not visit our institution at follow‐up for unknown reasons. The number of teeth present at follow‐up was reported in 655 women (follow‐up rate 77.7%).

### Data collection

Body height and weight were measured using standard procedures, and the body mass index (BMI) (kg/m^2^) was calculated. Patients who had received any treatment for osteoporosis and had diabetes mellitus (DM), dyslipidemia, hypertension, mild cognitive impairment, or a history of malignancy were included in this study. Current smokers and drinkers were included. Cognitive impairment was diagnosed using the Mini‐Mental State Examination.^(^ ^17^ ^)^ We excluded the patients with severe dementia. Except for osteoporosis medications, the number of drugs used, such as antihypertensive drugs, was assessed for all participants.

The primary endpoint during each observation period was final tooth extraction. The observation period was at least 1 year. The observation ended if the participants were unable to visit the primary‐care institution for treatment or if outpatient treatment was stopped because of any disease.

A physician trained in examining teeth recorded the number of teeth present at baseline and follow‐up. This physician also determined the presence of periodontal disease based on the methodology described in our recent study.^(^ ^16^ ^)^

### Diagnosis of fractures

Prevalent fractures were identified as nontraumatic. Each patient's history of long bone fractures was assessed during the interviews by a physician. Prevalent vertebral fractures were diagnosed based on a semiquantitative analysis of baseline radiographs of the thoracic and lumbar vertebrae (T_4_ to L_4_) using an established system.^(^ ^18^ ^)^

### Statistical analysis

Categorical variables are expressed as frequency (*n*) and percentage, and continuous variables are expressed as mean and standard deviation or standard error of the mean (SEM). The participants were divided into four categories: no prevalent fractures, prevalent vertebral fractures alone, prevalent nonvertebral fractures alone, and both prevalent fracture types.

First, a univariate analysis with a Poisson regression model adjusted for age was used to investigate the correlation between the number of teeth and certain variables. Next, a multivariate regression analysis adjusted for covariates that were significantly correlated with the number of teeth present at baseline was used to investigate the differences in the number of teeth present among the four categories. Data on periodontal disease in the edentulous participants at baseline were categorized as missing. Normalized transformation was applied to the categorical data on periodontal disease at baseline.

Similarly, univariate analysis with a Poisson regression model adjusted for age was used to investigate the correlation between the number of teeth lost and certain variables at follow‐up. Next, a multivariate regression analysis adjusted for covariates that were significantly correlated with the number of teeth lost was performed to investigate the differences in the number of teeth lost among the four categories. The Bonferroni adjustment was used for multiple comparisons in the Poisson regression analysis. All comparisons were two‐sided, and statistical significance was set at *p* < 0.05. Data were analyzed using the IBM SPSS software (version 24.0; IBM Corp., Armonk, NY, USA).

## Results

### Participant selection and characteristics

A total of 933 Japanese women were enrolled in this study. Among them, 899 were postmenopausal at baseline. Of these, the number of teeth was not recorded for 23 women, and data for one or more potential covariates were missing for 33 women. Ultimately, 843 participants were included in this cross‐sectional study. Follow‐up data on the number of teeth at the end of the observation period were available for the 655 women who were included in the longitudinal study.

The participant characteristics are presented in Table 1. Nonvertebral fractures without serious trauma were identified in 18 femurs, 34 distal ends of the radii, and 23 other areas (clavicle, fibula, pelvis, ribs, humerus, and tibia) at baseline.

**Table 1:** Characteristics of 843 Participants at Baseline and 655 Participants at Follow‐Up

			Mean (SD) or *n* (%)	
			Baseline	Follow‐up
Age (years)			68.3 (10.2)	67.4 (9.8)
Body mass index (kg/m^2^)			22.5 (3.3)	22.5 (3.4)
Drugs used (*n*)			3.2 (2.2)	3.2 (2.1)
Teeth present (*n*)			17.0 (10.2)	19.1 (8.6)
Teeth lost during follow‐up (*n*)				0.5 (1.2)
Toothbrushing sessions per day (*n*)			2.3 (0.8)	2.4 (0.8)
Follow‐up period (years)				4.0 (2.1)
Prevalent fractures (yes)		All	304 (36.1)	229 (35.0)
		PVF	229 (27.1)	169 (25.8)
		PNF	43 (5.1)	38 (5.8)
		Both	32 (3.8)	22 (3.4)
Diabetes mellitus (yes)			129 (15.3)	100 (15.3)
Dyslipidemia (yes)			435 (51.6)	341 (52.1)
Hypertension (yes)			479 (56.8)	372 (56.8)
Cognitive impairment (yes)			81 (9.6)	50 (7.6)
History of malignancy (yes)			122 (14.5)	99 (15.1)
Smoking (yes)			25 (3.0)	22 (3.4)
Alcohol use (yes)			82 (9.7)	62 (9.5)
Use of medications for osteoporosis (yes)			493 (58.5)	391 (59.7)
Periodontal disease	Yes		225 (26.7)	195 (29.8)
	No		514 (61.0)	460 (70.2)
	Missing		104 (12.3)	

*Note*: Data on periodontal disease in the edentulous participants were classified as missing.

Abbreviation: Both = prevalent vertebral and nonvertebral fractures; PNF = prevalent nonvertebral fractures alone; PVF = prevalent vertebral fractures alone.

The baseline characteristics of the study participants in the four fracture categories are shown in Table 2. A total of 539, 229, 43, and 32 participants had no prevalent fractures, prevalent vertebral fractures alone, prevalent nonvertebral fractures alone, or both prevalent fracture types, respectively. The prevalent nonvertebral fracture‐alone group included 24 (55.8%) distal radius fractures and 5 (11.6%) femur fractures. The group with both prevalent fracture types included 10 (31.2%) distal radial fractures and 13 (40.6%) femoral fractures.

**Table 2:** Characteristics of Participants in the Four Categories at Baseline

	NF	PNF	PVF	Both
Participants (*n*)	539	43	229	32
Age (years)	65.1 (0.4)	70.6 (1.3)	74.3 (0.6)	76.7 (1.4)
Body mass index (kg/m^2^)	22.5 (0.1)	22.6 (0.5)	22.5 (0.2)	22.6 (0.5)
Drugs used (*n*)	2.9 (0.1)	3.0 (0.3)	3.8 (0.1)	4.0 (0.4)
Teeth present (*n*)	18.8 (0.2)	19.5 (0.7)	12.5 (0.2)	14.1 (0.7)
Toothbrushing sessions per day (*n*)	2.34 (0.04)	2.35 (0.12)	2.01 (0.05)	2.41 (0.16)
Diabetes mellitus (yes)	81 (15.0)	9 (20.9)	38 (16.6)	1 (3.1)
Dyslipidemia (yes)	277 (51.4)	28 (65.1)	114 (49.8)	16 (50.0)
Hypertension (yes)	292 (54.2)	25 (58.1)	143 (62.4)	19 (59.4)
Cognitive impairment (yes)	38 (7.1)	3 (7.0)	35 (15.3)	5 (15.6)
History of malignancy (yes)	73 (13.5)	9 (20.9)	37 (16.2)	3 (9.4)
Smoking (yes)	21 (3.9)	1 (2.3)	3 (1.3)	0 (0)
Alcohol use (yes)	59 (10.9)	6 (14.0)	16 (7.0)	1 (3.1)
Medications for osteoporosis (yes)	243 (45.1)	30 (69.8)	191 (83.4)	29 (90.6)
Periodontal disease (yes)	136 (25.2)	15 (34.9)	67 (29.3)	7 (21.9)

*Note*: Data are shown as mean (SE of the mean) or *n* (%).

Abbreviation: Both = prevalent vertebral and nonvertebral fractures; NF = no fracture; PNF = prevalent nonvertebral fractures alone; PVF = prevalent vertebral fractures alone.

The characteristics of the study participants in the four fracture categories at follow‐up are shown in Table 3. Several characteristics, except for the follow‐up period and the number of teeth lost, remained the same as those at baseline. There were 426, 169, 38, and 22 participants with no prevalent fractures, vertebral fractures alone, nonvertebral fractures alone, or both prevalent fracture types, respectively.

**Table 3:** Characteristics of Participants in the Four Categories at Follow‐Up

	NF	PNF	PVF	Both
Participants (*n*)	426	38	169	22
Age (years)	64.5 (0.4)	70.7 (1.4)	73.0 (0.7)	75.6 (1.8)
Body mass index (kg/m^2^)	22.5 (0.2)	22.7 (0.5)	22.6 (0.3)	22.9 (0.4)
Drugs used (*n*)	3.0 (0.1)	3.3 (0.3)	3.6 (0.2)	4.1 (0.5)
Teeth at baseline (*n*)	20.6 (0.2)	19.2 (0.7)	15.2 (0.3)	20.5 (1.0)
Follow‐up period (years)	3.9 (0.1)	4.3 (0.4)	4.2 (0.2)	3.5 (0.4)
Teeth lost (*n*)	0.36 (0.03)	0.37 (0.10)	0.76 (0.07)	0.32 (0.12)
Toothbrushing sessions per day (*n*)	2.44 (0.04)	2.37 (0.13)	2.14 (0.06)	2.64 (0.19)
Diabetes mellitus (yes)	61 (14.3)	7 (18.4)	32 (18.9)	0 (0)
Dyslipidemia (yes)	227 (53.3)	24 (63.2)	80 (47.3)	10 (45.5)
Hypertension (yes)	233 (54.7)	24 (63.2)	102 (60.4)	13 (59.1)
Cognitive impairment (yes)	26 (6.1)	3 (7.9)	18 (10.7)	3 (13.6)
History of malignancy (yes)	64 (15.0)	7 (18.4)	25 (14.8)	3 (13.6)
Smoking (yes)	18 (4.2)	1 (2.6)	3 (1.8)	0 (0)
Alcohol use (yes)	44 (10.3)	5 (13.2)	12 (7.1)	1 (4.5)
Medications for osteoporosis (yes)	205 (48.1)	26 (68.4)	141 (83.4)	19 (86.4)
Periodontal disease (yes)	117 (27.5)	14 (36.8)	57 (33.7)	7 (31.8)

*Note*: Data are shown as mean (SE of the mean) or *n* (%). Except for the follow‐up period and number of teeth lost, most characteristics remained the same as those at baseline.

Abbreviation: Both = prevalent vertebral and nonvertebral fractures; NF = no fracture; PNF = prevalent nonvertebral fractures alone; PVF = prevalent vertebral fractures alone.

### Differences in the number of teeth present among the four groups at baseline

**Fig. 1:**
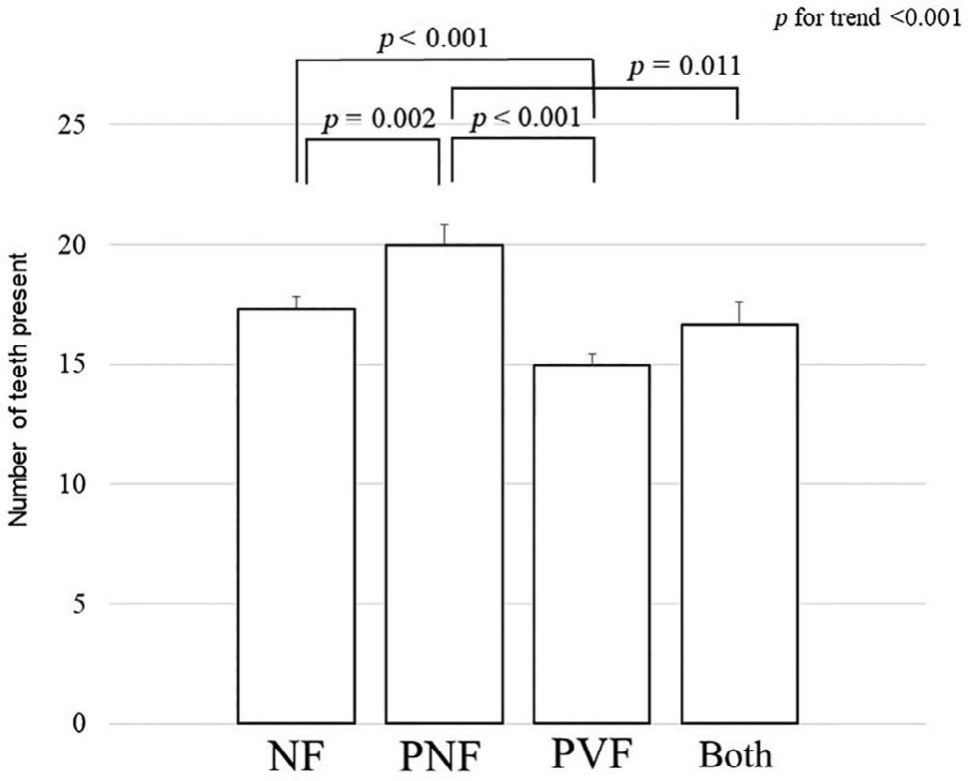
Number of teeth present (mean ± SE of the mean) in participants with no prevalent fractures (NF), prevalent nonvertebral fractures alone (PNF), prevalent vertebral fractures alone (PVF), and both types of prevalent fractures (Both) at baseline. Multivariate regression analysis adjusted for age, body mass index (BMI), number of drugs used, toothbrushing sessions per day, hypertension, cognitive impairment, history of malignancy, current smoking, alcohol use, medications for osteoporosis, and periodontal disease was applied to investigate differences in the number of teeth among the four categories. Participants with PVF had significantly fewer teeth (15.0 ± 0.5) than those with NF (17.3 ± 0.5) and PNF (20.0 ± 0.9) (*p* < 0.001 for both). Participants with PNF had significantly more teeth than those with NF (*p* = 0.002).

Univariate analysis at baseline revealed that the number of teeth present significantly correlated with age, BMI, number of drugs used, number of daily toothbrushing sessions, hypertension, cognitive impairment, history of malignancy, current smoking, alcohol consumption, use of osteoporosis medications, and periodontal disease (Table 4). The multivariate regression analysis adjusted for these covariates revealed that participants with prevalent vertebral fractures alone had significantly fewer teeth (mean ± SEM, 15.0 ± 0.5) than those in participants with no fractures (17.3 ± 0.5) and nonvertebral fractures alone (20.0 ± 0.9) (*p* < 0.001 for both) (Fig. 1). Participants with prevalent nonvertebral fractures alone had significantly more teeth than those in participants without fractures (*p* = 0.002).

**Table 4:** Univariate Analysis After Adjusting for Age at Baseline for Correlation Between Number of Teeth and Certain Variables

	Parameter estimate	SE of the mean	*p* Value
Age (years)	−0.026	0.008	<0.001
Body mass index (kg/m^2^)	−0.012	0.003	<0.001
Drugs used (*n*)	−0.041	0.004	<0.001
Toothbrushing sessions per day (*n*)	0.185	0.012	<0.001
Diabetes mellitus (yes)	0.022	0.023	0.337
Dyslipidemia (yes)	−0.016	0.017	0.352
Hypertension (yes)	−0.098	0.017	<0.001
Cognitive impairment (yes)	−0.181	0.035	<0.001
History of malignancy (yes)	0.083	0.024	<0.001
Smoking (yes)	0.112	0.043	0.009
Alcohol consumption (yes)	0.082	0.026	0.002
Medications for osteoporosis (yes)	0.052	0.018	0.003
Periodontal disease	−0.025	0.001	<0.001

*Note*: The results are presented as the parameter estimate, SE of the mean, and *p* value.

**Fig. 2:**
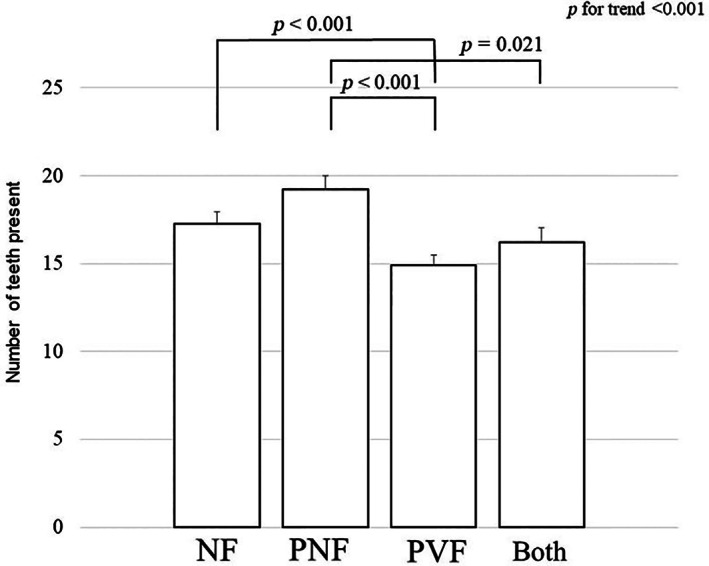
Number of teeth present (mean ± SE of the mean) in participants with no prevalent fractures (NF), prevalent nonvertebral fractures alone (PNF), prevalent vertebral fractures alone (PVF), and both types of prevalent fractures (Both) at baseline. Multivariate regression analysis adjusted for age, body mass index (BMI), number of drugs used, toothbrushing sessions per day, hypertension, cognitive impairment, history of malignancy, current smoking, alcohol use, medications for osteoporosis, and periodontal disease was applied to investigate differences in the number of teeth among the four categories. After additional adjustment for the history of distal radius fracture, the significant difference in the number of teeth present between participants with PNF and those with NF disappeared (*p* = 0.195), although other associations did not change.

In this study, ~56% of the prevalent nonvertebral fractures at baseline were located at the distal end of the radius. When the history of distal radius fractures was added to the multivariate regression analysis at baseline as a covariate, the significant difference in the number of teeth present between participants with prevalent nonvertebral fractures alone and those with no fractures disappeared (*p* = 0.195), although the other associations did not change (Fig. 2).

### Differences in the number of teeth lost among the four groups at follow‐up

**Fig. 3:**
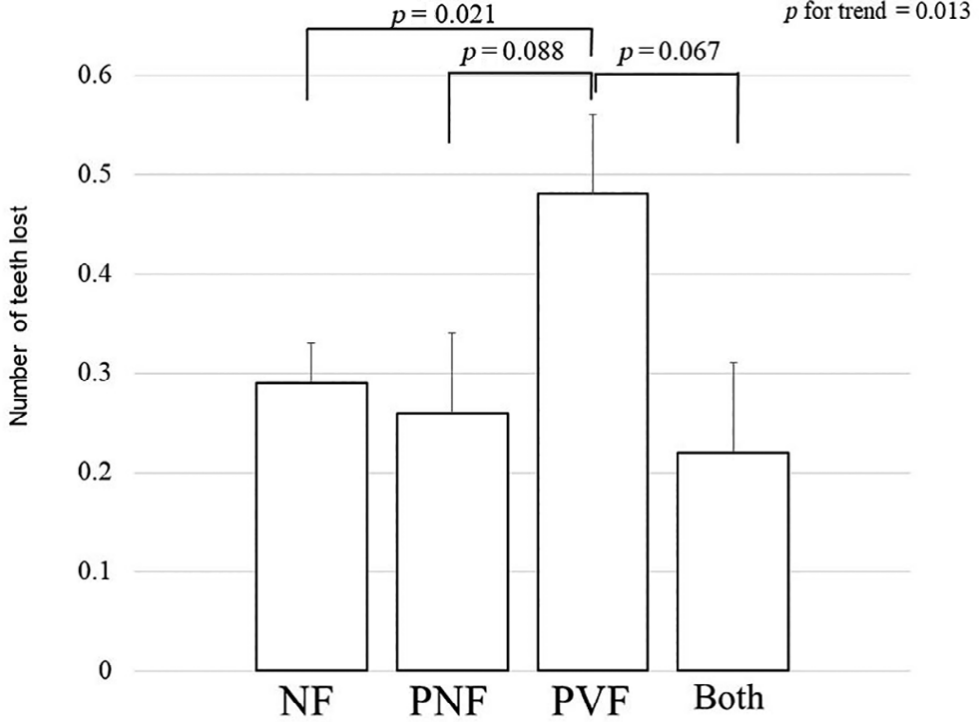
Number of teeth lost (mean ± SE of the mean) in participants with no prevalent fractures (NF), prevalent nonvertebral fractures alone (PNF), prevalent vertebral fractures alone (PVF), and both types of prevalent fractures (Both) at follow‐up. Multivariate regression analysis adjusted for age, number of teeth present at baseline, follow‐up period, hypertension, alcohol use, medications for osteoporosis, and periodontal disease was applied to investigate differences in the number of teeth lost among the four categories. Participants with PVF lost more teeth (0.48 ± 0.08) than those with NF (0.29 ± 0.04) (*p* = 0.021). Participants with PVF tended to lose more teeth than those with PNF (0.26 ± 0.08) and Both (0.22 ± 0.09) (*p* = 0.088 and *p* = 0.067, respectively). There was no significant difference in the number of teeth lost between participants with PNF and those with NF.

Univariate analysis at follow‐up revealed that the number of teeth lost was significantly correlated with age, number of teeth at baseline, follow‐up period, hypertension, alcohol consumption, use of osteoporosis medications, and periodontal disease (Table 5). Multiple regression analysis adjusted for these covariates revealed that participants with prevalent vertebral fractures alone lost more teeth (mean ± SEM 0.48 ± 0.08) than those in participants with no fractures (0.29 ± 0.04, *p* = 0.021) (Fig. 3). Participants with prevalent vertebral fractures alone tended to lose more teeth than those in participants with nonvertebral fractures alone (0.26 ± 0.08, *p* = 0.088) and both prevalent fracture types (0.22 ± 0.09, *p* = 0.067). There was no significant difference in the number of teeth lost between the participants with nonvertebral fractures alone and those with no fractures.

**Table 5:** Univariate Analysis After Adjusting for Age at the Time of Follow‐Up for Correlation Between Number of Teeth Lost and Certain Variables

	Parameter estimate	SE of the mean	*p* Value
Age (years)	0.020	0.006	0.001
Body mass index (kg/m^2^)	−0.016	0.018	0.360
Drugs used (*n*)	0.048	0.027	0.076
Teeth at baseline (*n*)	−0.039	0.007	<0.001
Follow‐up period (years)	−0.057	0.029	0.048
Toothbrushing sessions per day	0.104	0.070	0.139
Diabetes mellitus (yes)	−0.150	0.168	0.371
Dyslipidemia (yes)	−0.132	0.116	0.254
Hypertension (yes)	0.329	0.124	0.008
Cognitive impairment (yes)	−0.059	0.213	0.782
History of malignancy (yes)	0.086	0.154	0.578
Smoking (yes)	−0.825	0.505	0.103
Alcohol use (yes)	−0.706	0.275	0.010
Medications for osteoporosis (yes)	0.353	0.130	0.006
Periodontal disease (yes)	0.776	0.115	<0.001

*Note*: The results are presented as the parameter estimate, SE of the mean, and *p* value. Except for the follow‐up period, most characteristics remained the same as those at baseline.

## Discussion

To our knowledge, this was the first study to investigate the association between prevalent vertebral fractures and the number of teeth present at baseline and that lost during the follow‐up period. In this study, postmenopausal Japanese women with prevalent vertebral fractures alone had significantly fewer teeth at baseline and lost more teeth during follow‐up than did those without fractures. Several studies have reported an association between skeletal BMD and the number of teeth in postmenopausal women.^(^ ^6^, ^7^, ^19^, ^20^, ^21^, ^22^, ^23^ ^)^ Southard and colleagues reported significant correlations between the maxillary alveolar bone density and anteroposterior lumbar spine (*r* = 0.53, *p* < 0.001), lateral lumbar spine (*r* = 0.52, *p* < 0.001), total hip (*r* = 0.39, *p* = 0.01), and total radius (*r* = 0.39, *p* = 0.01) bone densities.^(^ ^24^ ^)^ We previously reported that the odds of low spinal BMD in participants with self‐reported periodontal disease were 2.01 (95% confidence interval [CI] 1.15–3.50) in 253 early postmenopausal Japanese women.^(^ ^25^ ^)^ Periodontal disease may progress in postmenopausal women with a low spinal BMD, resulting in an increased risk of tooth loss. In addition to the similarity in two‐dimensional BMD, the bone architecture of the vertebral body and alveolar bone are likely to be similar, consisting of a trabecular bone surrounded by a thin cortical bone. We previously demonstrated a significant association between mandibular and lumbar vertebral trabecular BMDs measured using quantitative computed tomography in postmenopausal Japanese women (*r* = 0.41, *p* < 0.05).^(^ ^26^ ^)^ Thus, architectural damage to the vertebral body and alveolar bone supporting the teeth may progress simultaneously.

The reason for increased tooth loss in postmenopausal women with prevalent vertebral fractures remains unclear. Martínez‐Maestre and colleagues found that postmenopausal women with prevalent spinal fractures lost more teeth and had more advanced attachment loss than did participants without fractures.^(^ ^15^ ^)^ However, the prevalence of mild and moderate periodontal attachment loss was not significantly different among the groups. They suggested that clinical periodontal differences between postmenopausal women with and without osteoporosis could only be detected in the advanced stages of both diseases. Spinal fractures and tooth loss may occur in the advanced stages.

In previous epidemiological studies, the main outcome of osteoporosis was self‐reported fractures. However, this approach is unsuitable for vertebral fractures because they are often clinically asymptomatic.^(^ ^27^ ^)^ Therefore, regular evaluation of vertebral radiographs is necessary to accurately determine the presence of vertebral fractures. However, it is difficult to assess vertebral fractures epidemiologically among community residents. In this study, we obtained vertebral radiographs at baseline and every 1 to 2 years to assess the prevalence and incidence of vertebral fractures in patients visiting a primary‐care institution. To our knowledge, this was the first study to observe the relationship between vertebral fractures and tooth loss.

In this study, participants with prevalent vertebral fractures alone lost more teeth during the follow‐up period than did those without prevalent fractures. Because the mean age of our participants was 68.3 years at baseline, those with fewer teeth may have wished to retain as many teeth as possible. Moreover, women with more teeth at the beginning of the study may have retained more teeth. However, even in the relatively short follow‐up period (mean 4.0 years), a significant difference was observed in the number of teeth lost between participants with prevalent vertebral fractures alone and those without fractures. Similar architectural damage to the vertebrae and alveolar bone may have contributed to the tooth loss at follow‐up. Furthermore, the number of brushing sessions per day was lower in participants with prevalent vertebral fractures alone (mean ± SEM 2.14 ± 0.06) than in those with no fractures (2.44 ± 0.04), as shown in Table 3. This may also be related to the increased risk of tooth loss during follow‐up in the participants with prevalent vertebral fractures. Conversely, the number of teeth lost and daily toothbrushing sessions were similar between participants with nonvertebral fractures alone and those without fractures. Therefore, the fragility of the nonvertebral cortical bone could be independent of the deterioration of the alveolar bone, which contributes to tooth loss.

In this cross‐sectional study, participants with prevalent nonvertebral fractures alone had significantly more teeth than those in individuals without fractures (*p* = 0.002). However, after additional adjustment for a history of distal radius fracture, which accounted for 56% of nonvertebral fractures alone, this association disappeared. Tsukutani and colleagues reported that the highest incidence of distal radial fractures was observed in participants aged 60 to 69 years, whereas hip fractures were primarily observed in those aged ≥85 years.^(^ ^28^ ^)^ Because distal radial fractures occur in relatively younger postmenopausal women, patients with distal radial fractures may start taking medications for osteoporosis at a younger age than do those with other nonvertebral fractures, such as hip fractures. Estrogen therapy has a protective effect against tooth loss.^(^ ^9^, ^10^, ^11^ ^)^ Penoni and colleagues described that bisphosphonate therapy improved bone fragility, periodontal status, and tooth loss in women aged 65 to 80 years.^(^ ^29^ ^)^ Our recent study suggested that once‐yearly zoledronic acid combined with regular oral health care may effectively prevent tooth loss in postmenopausal women.^(^ ^30^ ^)^ The effect of the early prescription of medications for osteoporosis to prevent distal radial fractures might have contributed to the higher number of teeth in participants with prevalent nonvertebral fractures than in those without fractures at baseline in this study.

This study had some limitations. Our participants were not entirely representative of postmenopausal Japanese women. Of the 843 participants, 371 (44.0%) used osteoporosis medications. Previous studies have suggested osteoporosis medications display protective effects against tooth loss in postmenopausal women.^(^ ^9^, ^10^, ^11^, ^29^, ^30^ ^)^ Considering these reports, the osteoporosis medications might have influenced our results. We adjusted for the use of osteoporosis medications in the analysis; however, no information was collected on specific details such as the duration of medication use or the combination of agents. This study included only women; therefore, the findings should be confirmed in men. Furthermore, the large difference in the number of participants between the groups may have influenced the results of this study.

In this study, physicians counted the number of teeth and determined the presence of periodontal disease. The presence of dental implants was confirmed before teeth were counted. Because this physician was well trained in counting teeth, miscounting at baseline and follow‐up was unlikely. Each participant was interviewed individually to evaluate periodontal status, including gingival bleeding, swelling, and/or pain. Dentists usually diagnose periodontal diseases using precise periodontal indices. However, the quality of the collected information depends largely on examiner competence.^(^ ^31^ ^)^ The difficulty of examiner alignment, especially in large‐scale studies, may result in considerable disagreement when determining periodontal disease.

In the present study, the potential risk factors associated with tooth loss, such as obesity,^(^ ^32^ ^)^ DM,^(^ ^33^ ^)^ dyslipidemia,^(^ ^34^ ^)^ hypertension,^(^ ^35^ ^)^ and cognitive impairment,^(^ ^36^ ^)^ were evaluated. However, low oral health awareness, such as during routine dental checkups, may be one of the predictors of tooth loss in older Japanese.^(^ ^37^, ^38^ ^)^ Further studies that evaluate additional risk factors are necessary to confirm our findings.

This study was unable to confirm the type of tooth extracted, such as an incisor, canine, premolar, or molar, from the maxilla and/or mandible. Information on the type of tooth extracted may clarify why prevalent vertebral fractures are associated with an increased risk of tooth loss in postmenopausal women. Dental radiographs are necessary to gather this information in future studies.

In conclusion, prevalent vertebral fractures may be associated with an increased risk of tooth loss in postmenopausal Japanese women. Postmenopausal women with prevalent vertebral fractures should be referred to dental clinics and undergo regular oral health care to prevent tooth loss.

## Author Contributions

**Akira Taguchi:** Conceptualization; formal analysis; funding acquisition; investigation; methodology; resources; validation; writing – original draft; writing – review and editing. **Tomohiko Urano:** Formal analysis; supervision; visualization; writing – review and editing. **Yukio Nakamura:** Formal analysis; supervision; visualization; writing – review and editing. **Masataka Shiraki:** Data curation; formal analysis; funding acquisition; investigation; methodology; project administration; resources; software; visualization; writing – original draft; writing – review and editing.

## Disclosures

All authors declare that they have no conflicts of interest.
